# OPC UA Publish-Subscribe and VSOME/IP Notify-Subscribe Based Gateway Application in the Context of Car to Infrastructure Communication

**DOI:** 10.3390/s20164624

**Published:** 2020-08-17

**Authors:** Alexandru Ioana, Adrian Korodi

**Affiliations:** Department of Automation and Applied Informatics, Faculty of Automation and Computers, University Politehnica Timisoara, 300223 Timisoara, Romania; tm.alexandru@yahoo.com

**Keywords:** gateway application, OPC UA Publish-Subscribe, VSOME/IP Notify-Subscribe, automotive, car to infrastructure communication, communication protocols

## Abstract

With the recent advances in the area of OPC UA interfacing and the continuously growing requirements of the industrial automation world, combined with the more and more complex configurations of ECUs inside vehicles and services associated to car to infrastructure and even car to car communications, the gap between the two domains must be analyzed and filled. This gap occurred mainly because of the rigidness and lack of transparency of the software-hardware part of the automotive sector and the new demands for car to infrastructure communications. The issues are related to protocols as well as to conceptual views regarding requirements and already adopted individual directions. The industrial world is in the Industry 4.0 era, and in the Industrial Internet of Things context, its key interfacing enabler is OPC UA. Mainly to accommodate requirements related, among others, to high volumes, transfer rates, larger numbers of nodes, improved coordination and services, OPC UA enhances within its specifications the Publish-Subscribe mechanism and the TSN technology. In the OPC UA context, together with the VSOME/IP Notify-Subscribe mechanism, the current work is stepping toward a better understanding of the current relation between the needs of the industry and the suitable technologies, providing in-depth analysis on the most recent paradigms developed for data transmission, taking in consideration the real-time capabilities and use-cases of high concern in automation and automotive domains, and toward obtaining a VSOME/IP—OPC UA Gateway that includes the necessary characteristics and services in order to fill the protocol-related gap between the above mentioned fields. The developed case study results are proving the efficiency of the concept and are providing a better understanding regarding the impact between ongoing solutions and future requirements.

## 1. Introduction

### 1.1. The Context of Car to Infrastructure Communication

The current conceptual approaches in the context of Industrial Internet of Things (IIoT) are determining the directions of the industry.

In the context of car to infrastructure, intra-car and inter-car communication necessities, several strategies are approached. The strategies are focusing on the following directions: (a) Increasing the data volumes, transfer rates, reliability, and security of protocols; (b) developing gateway solutions for the best defined protocols to unify the worlds of cars (automotive) and infrastructure (manufacturing), and to translate information in the intra-car environment between different hierarchical control levels; (c) centralizing the infrastructure data and the cars data to provide solutions and answers mainly for the cars. Obviously, the most relevant and intriguing from a scientific point of view and also from the practical perspective are the first two mentioned strategies. The main scope is to ignition a smart infrastructure direct secure communication with the smart vehicles. A common ground regarding protocols and solutions has to be found between the continuously developing strategies in the industry that will smarten the infrastructure [1,2,3], respectively the automotive in-car communications that are not so dynamic [4].

In the automotive field, both automotive manufacturing and in-car developments, regarding the traffic control, the current developments are focusing on centralized and integrated control of the dynamic environment using a management model. According to [5], research is being carried out to be able to remove traffic lights from smart cities, identifying and interpreting traffic signals being the goal of autonomous vehicles. Industries and research groups still face the active problem of detecting and interpreting traffic lights. Also, in [6], the authors are mentioning the necessity of genetic algorithms for in-car systems for detecting and recognizing traffic lights, including identifying problems such as partial occlusions and inherent LED failures of autonomous systems.

The model of the traffic light control system has certain characteristics for a correct and safe operation: the controller is not allowed to block (deadlock) because of unexpected combinations of actions; conflict movements should not have the right of way at the same time; it should be able to serve all phases of the signal and return to a certain initial state. As mentioned in [7], to avoid collisions and other hazards, the drive is relying on a set of rules and instructions set by the traffic light controller. At the base of traffic control systems are signs, lights, and other devices that communicate specific indications, warnings, or requirements. In [7], the traffic light controller (TLC) was implemented using microcontroller, Field-Programmable Gate Array (FPGA), and Application Specific Integrated Circuit (ASIC) design. The drive in the modern world, as mentioned in [8], means both normal and self-driving in the network traffic, using high-definition maps, real-time updates, multimedia, etc.

Introducing Ethernet requires a gateway to existing automotive network technologies for smooth migration, as mentioned in [9]. Premium cars have more than 80 ECUs and the demand for inter-ECU communication has increased greatly. In the current vehicle architecture, the gateway is the only device that ensures network communication between different networks. The gateway is one of the most important components for distributed applications and must assure that messages transmitted over the network meet their constraints.

The interest for intelligent cars has increased to reduce the possibility of accidents providing traffic safety services. Smart car is a vehicle that offers safety and combines electronic technology and IT technology with old automotive technology to recognize the safety status of the internal or external car area. A lot of embedded systems are implemented into the vehicle offering safety of drivers and pedestrians. Vehicle-to-Everything (V2X) communication technology connects vehicles to interact with each other Vehicle-to-Vehicle (V2V), road infrastructure Vehicle-to-Infrastructure (V2I) through wireless communications. V2X is a communication technology that connects vehicles to the road. Connected vehicles interact with each other (V2V), road infrastructure (V2I) through wireless communications. For safety, V2I communication represents the exchange of critical safety and operational information between vehicles and road infrastructure, mainly intended to prevent or reduce vehicle crashes, but also to enable a wide range of safety measures. V2I communications apply to any type of vehicle and road and transforms infrastructure equipment into smart infrastructure. According to [10], the control center collects safety information, such as car accidents, traffic jams, bad weather using Long-Term Evolution (LTE), Closed-circuit television (CCTV), Global Positioning System (GPS) devices in the respective regions. If traffic incidents occur in the specific area, it triggers the CBC (Cell Broadcast Center) control to transmit the information that the security system has to start. The gateway is based on two separate Multipoint Control Units (MCUs) on the same board, as the AUTOSAR software runs on top of the AUTOSAR OS system which is optimized for the vehicle’s E/E systems. Therefore, one MCU (MCU1) is to load the LTE module and the application to transpose the LTE data in the event on the vehicle, and the other (MCU0) is to run the AUTOSAR platform and application to trigger appropriate security systems. If an accident happened in an area where the sight is not good, then the infrastructure sends the information to vehicles. This information may include accident information, such as distance, lane, and speed in the respective area. With this information, the gateway triggers the security system in the vehicle. Automotive technology must be cautious in adopting new systems, as the vehicle is directly involved with human safety. In [11], a multi-protocol gateway is implemented that supports three popular communication protocols, the Controller Area Network (CAN bus), WI-FI and Recommended Standard 232 (RS-232). The gateway is implemented on an embedded Linux system ported to an Advanced RISC Machine (ARM) development board. A dedicated application is designed to handle the exchange of information between gateway ports. The Access Control List (ACL) is implemented to control the permission of data frames on the CAN bus. It is considered useful where the GPS signal is not available, for example in a tunnel. Because of the increased number of approaches regarding connected and autonomous vehicle applications, studies as [12] are analyzing risk models for assessing intelligent transportation systems devices. Studies as [13] are proposing deep reinforcement learning for left-turn in the case of connected and automated vehicle control at signalized intersections in vehicle-to-infrastructure environment, focusing on centralizing data. Authors in [14] underline that application software for the automotive industry is becoming more and more needed. According to [14], there are car manufactures like Daimler AG or BMW that have already upgraded wireless connectivity solutions to the latest automotive infrastructure, such as attaching devices with Wi-Fi or Global System for Mobile communication (GSM) features, which offer high connectivity based on 3G/LTE Technologies and locally, through Wi-Fi access points. The software solution is represented by Remote Diagnosis Application. The UNIX distribution used is a dedicated real-time operating system, meaning that it is capable of meeting time constraints and data consistency, a general requirement for the automotive industry. Some automatic recovery mechanisms are implemented by the software environment in real time, for example the “watchdog” mechanism.

### 1.2. Emerging Protocols

The Open Platform Communications Unified Architecture (OPC UA) protocol (IEC 62541) is the main enabling interface for Industry 4.0 and IIoT. Therefore, it is a key feature for the industry that manufactures everything related to the infrastructure. In [15] the author presents the status of the automotive industry including traffic signals exposing OPC UA as the recommended protocol in car to car and car to infrastructure interfacing. As described in [16], connecting electronic devices equipped with fieldbus communication capabilities in emerging Cyber Physical Systems (CPS), involves the creation of new models that combine live data from the underlying devices with metadata information describing them. The new solutions must be based on widely accepted communication standards and should be easy to implement even in the case of limited resources. The authors are expressing the necessity to convert intra-car CAN-based systems into OPC UA. Work [16] focuses on an OPC UA server used as a gateway to share information available on a control network (CAN), implemented using the C# SDK from Unified Automation. It links live data from physical devices with meta information that support their correct understanding. Engineering knowledge is taken directly from CANoe which is used to develop CAN-based systems. The information is available to any OPC UA Client according to the OPC UA data model. The address space is based on the client-server paradigm and the CAN communication used for addressing messages and multicast communication. The CAN handles the data from the physical device, but it is not suitable for information transmission, respectively, the meta information about a CAN-base system is not visible in the CAN network, it is available only in the development environment. For an efficient exchange of information, a secure connection must be set between the Client and the Server. The client can search or browse in the address space of the data server. The OPC UA address space can include many servers and clients. If part of the information is stored on another server, a connection is made by references. The connection made in a certain way called subscription helps to determine the important information for the client. Such a communication model allows for flexible and efficient communication in open systems. In [16], the authors are not defining the approach toward the latest OPC UA specifications. The authors in [17] are analyzing the in-vehicle communication strategies and are presenting a perspective toward new protocols. OPC UA is considered a future protocol following the experience of the manufacturing industry. The given perspective, without providing a final grounded solution, is either for in-vehicle data exchange or as a conversion and parking solution to interface the CAN with external devices.

As mentioned in [18], modern vehicles use a large amount of distributed computing and require a basic communication scheme to provide high bandwidth and low latency. The communication protocols, Controller Area Network (CAN) and FlexRay, do not provide the required bandwidth, so adopting Ethernet as the next generation network will become the backbone for in-vehicle systems. In [18], the authors state that a high-performance network gateway can simultaneously handle high bandwidth, low latency, and isolation and the mentioned features cannot be obtained with traditional processor-based gateway implementations. Also, the large number of electronic control units (ECUs) involved in data processing and driver assistance have to include time and safety critical functions. The information from these ECUs would be gathered in a central gateway, and the authors from [18] mention that the communication with ECUs from other domains should be realized using these central gateways. Also, it is mentioned that Ethernet is expected to be for the next generation vehicle architectures, the backbone of the network, while the CAN, Local Interconnect Network (LIN), and FlexRey networks continue to support the respective application classes. MCU-based gateways allow the exchange of reliable messages for traditional networks, operating at 1–10 Mbps, but they do not meet the data rates of 100 Mbits/s or higher of Ethernet networks.

According to [19], vehicles are becoming more intelligent, connected, and part of the Internet. While new features are being developed, such as natural speech recognition and cloud-based services, legacy systems in the vehicle need to be supplemented with new developments to shake up cost efficiency. Thus, traditional signal-based communication, consisting of cyclic message transmissions, such as in LIN, CAN/CAN-FD, and FlexRey, must coexist with service-based communication, consisting of event-based unicasts, such as IP-based networks. The scalable service-oriented IP intermediary allows for the introduction of service-oriented information transmission, where a sender only transmits data when at least one network receiver needs this data, avoiding the network load and all nodes connected to useless traffic. Also in [19], the authors state that the dynamic distribution of functions, the virtualization of the ECUs, and the network controlled by the virtual machine and the network hypervisor are in the future car network architectures, which are currently migrating from the current structure of the central gateway to a domain-based architecture. It will follow that the Input/Output (I/O) vehicle architecture will be characterized by a car Ethernet backbone that connects different domains, isolated and protected by domain controllers. The Ethernet central switch will be connected to a smart antenna, being LTE/5G, Wi-Fi/BLE, and V2X/DSRC. After developing the Institute of Electrical and Electronics Engineers (IEEE) standards within the Time-Sensitive Networking (TSN) Working Group, the vision for the automotive Ethernet backbone in which different domains connect, each with its TSN control unit, provides master MCUs that integrate Ethernet PHYs and TSN switches that have security modules and various protocol converters for legacy local serial networks. Authors in [20] are presenting a Long Rage (LoRa)-based physical layer key generation for secure vehicle to vehicle and vehicle to infrastructure communications, without detailing other layers on the Open Systems Interconnection (OSI model).

Although not exposed much in literature, now the automotive industry is considering the Scalable service-Oriented MiddlewarE over IP (Some/IP) protocol as being the higher-level protocol for in-car ECUs, VSOME/IP being Genivi’s Some/IP. Some/IP is also considered by the slowly moving automotive industry as the next generation communication protocols between local ECUs. As detailed above, OPC UA is foreseen as a prospect that can replace Some/IP. But the current prospect would be to consider a gateway for the Some/IP and OPC UA conversion and wrapping.

OPC UA recent specifications are referring explicitly systems that are requiring high volumes of data transfer, fast responses, and real time. In this context, the work [21] is considered very significant, as it approaches the OPC UA TSN (Time Sensitive Networking) technology by providing an evaluation of the applicability of OPC UA TSN in factory automation. Also, [22] is presents a study related to TSN regarding the field devices. Considering more and more OPC UA TSN enabling studies and the big players from the industry joining the previously mentioned concepts, the transition to automotive is imminent.

In the above-mentioned context and the new technological researches especially regarding OPC UA, the current paper extends and improves the conceptual approach of the VSOME/IP—OPC UA Gateway presented in [23]. The new approach focuses on:

- The development of a VSOME/IP—OPC UA Gateway in the context of a detailed analysis and development of the OPC UA Publish-Subscribe mechanism with respect to the TSN technology and considering the OSI model;
- Presenting the necessary steps to implement the OPC UA Publish-Subscribe mechanism and to be tested together with the VSOME/IP Notify-Subscribe mechanism using a VSOME/IP—OPC UA gateway experimental model;
- Analyzing and implementing the real-time behavior on the application level through synchronizing some processes associated with the involved entities;
- Providing a clear view over some intensively stated new concepts that will be of high importance in the vehicle to infrastructure context.

## 2. Materials and Methods

The chapter discusses in detail concepts as TSN, OPC UA over TSN, OPC UA Publish-Subscribe mechanism, the real-time perspective in OPC UA, that are very much referred currently but most times not well grounded. Also, it details the new approaches regarding the developments associated to the Gateway application, focusing on OPC UA. The chapter is also meant to discuss various aspects that are considered filling the knowledge gap between the automotive in-car hardware-software development industry and generally the manufacturing industry.

### 2.1. Time-Sensitive Networking Context

Time-sensitive networking (TSN) represents a solution for a time-controlled transfer of real-time critical messages over standard hardware. In recent years, the classic communication over Ethernet has begun to be used on controller to controller communication and on sensors to cloud communication, in applications with real-time constraints. With Ethernet being part of many high level communications protocols, with applications running on different hardware and with different networks with various capabilities being involved in the communication flow, the need of a network technology which could guarantee time sensitive messaging as part of the infrastructure for applications that implement Ethernet based communication protocols, was only a matter of time.

TSN is based on a series of individual standards, which mainly concern the datalink layer (layer 2 on the OSI model) of communication. TSN should not be classified as a communication protocol, but rather as a basic technology, which can then be used by Ethernet-based communication protocols (as OPC UA) in use-cases with real-time requirements.

TSN main features over the existing Raw Ethernet infrastructures are the time synchronization, low latency times, and seamless redundancy. The application software will transmit network-specific requirements regarding priority, time cycles for different operations, and specific configuration data, and the network will make sure that all implied services will guarantee the delivery of the network messages accordingly to the application needs. TSN also makes possible for a network to be convergent, different real-time protocols being able to communicate and exchange information inside a single network, offering unreachable flexibility with different infrastructures, making the investment in such an infrastructure to be profitable in distant future.

Sensitive network technology is composed of different Ethernet sub-standards defined in the IEEE 802.1 TSN Task Group. The main goal of standardizing parts of the Data Link Layer is not yet fully fulfilled, with upgrades and changes still possible to come, but even so, the main features can be integrated in products and applications.

With multiple sub-standards being involved in the TSN technology, and the main goal being the real-time functionality of the network, it is essential to mention some of the most important of them. For achieving the main goal, all equipment connected to the same network must have a common time reference by which the general synchronization of the entities inside the network is made. The IEEE 1588-2008-Precision Time Protocol provides the Grandmaster Clock (the most accurate clock from the participants) of the network, using algorithms within the network. The IEEE 802.1AS-2011—Time protocol manages data traffic with guaranteed delivery time. For the prioritization of critical and non-critical data, the IEEE 802 1Q standard is establishing 8 traffic classes. Because of the buffer mechanism of the Ethernet switches, packets with low priority can produce delays for the critical data, so additional mechanism are necessary for increased efficiency regarding the priority and scheduling of the data streams. One of these mechanisms is the Credit Based Shaper (IEEE 802.1Qav). Being developed for the Audio/Video Bridging technology, a predecessor for the current TSN technology, the Credit Based Shaper is in charge of assigning send credits to data streams, credits spend during transmission as long as the value is positive. The mechanism promotes the principle of best effort traffic, so when a transmission runs out of send credits, the best effort data packets next in line are transmitted. If this process delays the transmission of packets with higher priority, the value of the send credits is increased, so the next packets of data with high priority will be transmitted as soon as the traffic allows it.

Another mechanism in charge of increasing efficiency of the transmission of critical-data is the Time-Aware Scheduler (IEEE 802.1Qav). Similar to the scheduler mechanism present in applications with real-time requirements, the Time Aware Scheduler creates cycles of discrete time on which the traffic classes, based on their priority, can send data at established moments in time. Owing to this type of scheduler, the transmission and receiving of data streams can be predicted for different types of messages, synchronization of multiple transmissions is easier, and all entities connected to the network are aware of the priority of the message, when the message is sent and when the message should be received and processed.

For applications with real-time requirements involved in certain use cases, the TSN technology provides flexibility by adapting different standards to different scenarios, not all mechanisms are needed to exist if the network can fulfil all requirements, so the TSN network can be configured to match the costs and efficiency expectations for each type of applications.

### 2.2. OPC UA Over TSN

With the development of Industrial Internet of Things (IIoT) in the last years, the need of information exchange at a higher rate and with different participants in the process, have generated more complex scenarios and more demanding requirements for applications in the field. It was expected for the high-level communication protocols to expand the possibilities, and to adapt and interact with different, more capable technologies even on lower levels of the OSI model. The production processes, the existing infrastructure and the production lines will also need to evolve, IIoT’s commitment is to increase efficiency in the existing processes, targeting also flexibility, profitability, and continuous maintenance of future developed products.

Extension of the OPC UA reaches down to the field level, cloud interaction, controller to controller communication, integration with different message brokers, easy and cheap solutions for large scale applications implying high volume of information transmission, artificial intelligence algorithms present in IoT products, are only some of the use-cases for which real-time requirements, synchronization, low latency, and vendor independent hardware, are demanded, so interaction between OPC UA and a technology with capabilities to fulfill such requirements like TSN, represents the next step in the automation field.

OPC UA over TSN expand the possibilities of the current technology by altering the automation model, with hierarchy between enterprise level, process management, control, and field buss level into a new model where the connectivity inside the network will be available at higher scale, between different participants, many production processes being achieved and improved with less cost and more flexibility. The OPC UA capabilities and the improvement of the lower layers using TSN (e.g., Data Link Layer) achieve a wider approach in terms of network proficiency, architecture, and business strategy in the context of Industry 4.0.

### 2.3. OPC UA Publish-Subscribe Mechanism in the TSN Context

In order for OPC UA to evolve and improve automation in the context of Industry 4.0, for achieving real-time functionality and integration of standard vendor independent hardware with fast communication demands, and also integration in cloud-based infrastructures, the OPC Foundation designed the Publish-Subscribe standard as part 14th of the OPC UA Specifications [24]. When discussing field level communication and controller to controller communication, the needs are represented by low latency in one-to-one transmission. When discussing cloud-based transmission, the context changes in one-to-many or many-to-many transmissions which increase the capabilities of the network. When combining all patterns and different types of devices at large scale, the Publish Subscribe mechanism proves to be suitable in terms of flexibility, performance, and based on the chosen type of transport, permissive on integrating devices with different capabilities in the same context.

**Figure 1:**
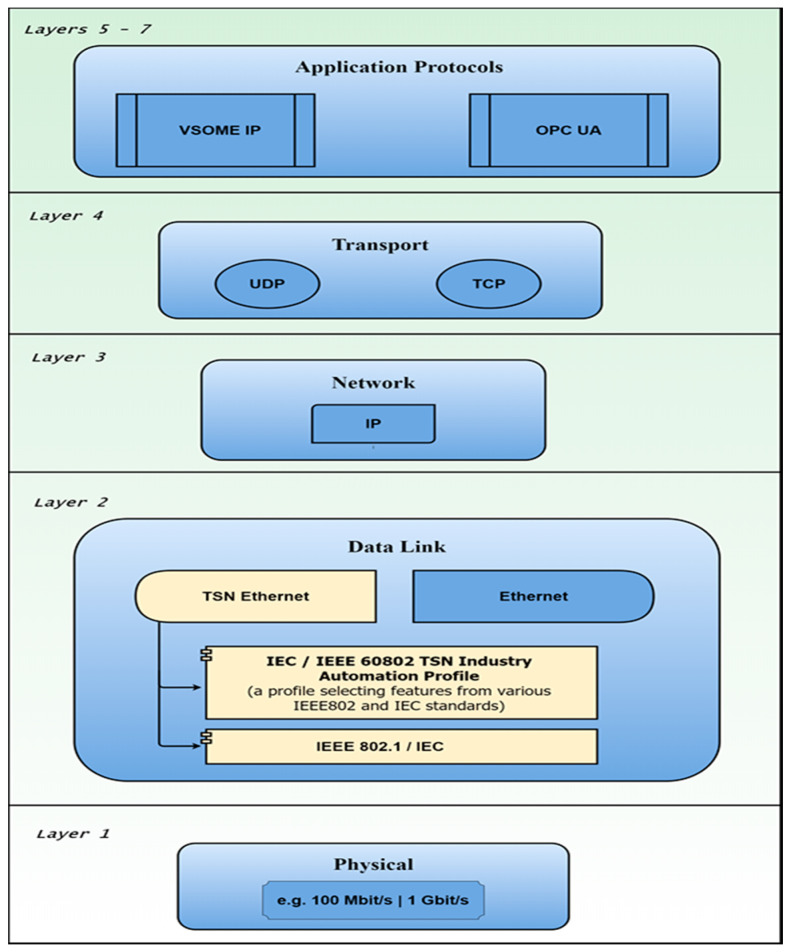
OPC UA and VSOME/IP application protocols in relation UDP, TSN, Raw Ethernet, in the context of the OSI layers.

Based on the OPC Unified Architecture Part 14: PubSub (chapter 4.2—see [24]), Publish and Subscribe can use at transport level, different types of protocols based on the information type and network capability. For frequent transmissions and low information volume, UDP transport with binary encoded messages represents a good solution in one-to-one and one-to-many transmissions. For cloud integration and analytics-based applications, standard messaging protocols like MQTT and AMQP are preferred, providing integration of different devices as subscribers on the Middleware level of the Publish Subscribe pattern. Based on the flexibility and easy device integration, real-time requirements must be resolved, and TSN technology creates the possibility of achieving the quality of service regarding synchronization, speed and predictability, and bandwidth-protection, by implementing different Ethernet-specific standards targeting the Data Link Layer, expanding capabilities of the OPC UA in scale and capacity. In the context of relating the concepts, the specifications and the protocols approached by the current study, Figure 1 depicts the relation with respect to the OSI layers of the OPC UA and VSOME/IP application protocols, with the transport layer (e.g., UDP), the network layer, the data link layer, and the physical layer.

### 2.4. OPC UA Publish-Subscribe Approach and Implementation

For implementing a Publisher and a Subscriber, the first step is to create a connection for both entities. As the OPC Unified Architecture Part 14: PubSub mentions (chapter 6), Publish-Subscribe defines different configuration parameters for different components. Based on the configuration, the publisher or the subscriber will gain certain behavior. The Publish-Subscribe connection deals with details regarding the transport protocol. Other subcomponents of the Publish-Subscribe connection are the WriterGroup and the ReaderGroup and each connection can have more than one group. The WritterGroup and the ReaderGroup contain details necessary for creating DataSetMessages.

The DataSetMessages are specific to other subcomponents, called DataSetWritter for the WritterGroup, respectively DataSetReader for the DataSetReader group. The DataSetWritter is the entity responsible of creating DataSetMessages. Based on the type of the information, the DataSetMessage can have multiple fields. After the DataSetWritter is created, it is bound to only one PublishedDataSet. A PublishedDataSet can be seen as the embodiment of all necessary parameters describing the content of a Message. However, the PublishedDataSet can have multiple DataSetWriter entities.

In a context in which the Publisher sends information and the Subscriber want to access that information, the DataSetReader will be part of the Subscriber application. The Subscriber receives, network messages, that along with protocols-specific information based on the transport type and network specific data. For obtaining the payload, the target information, the network message must be filtered based on the configurations used by the Publisher before being sent. This responsibility comes to the DataSetReader which extract the useful information from the network message and at this moment, the payload can be accessed field by field by browsing through different fields if the payload represents values of variables with different data types, or by reacting to different types of events.

The Network Messages must not be confused with DataSetMessages which signify the payload. The Network Messages are indeed containing the payload provided by the Publisher, but they are specific to a Middleware between Publisher and Subscriber, and are different in terms of encoding based on the preferred transport protocol.

The Subscriber receives Network Messages, respectively through filters and with the configured DataSetReader component, the published payload is obtained. The middleware can be represented by a broker in cases of protocols like Message Queuing Telemetry Transport (MQTT) and Advanced Message Queuing Protocol (AMQP), or by a multicast address when using User Data Protocol (UDP).

The open62541 SDK ([25]) was used for the current study to implement all OPC UA entities and Genivi’s version of Some/IP protocol (VSOME/IP) have been used to implement all VSOME/IP entities.

### 2.5. Real-Time Context—What Real-Time Should Mean

The real-time concept is largely used in different fields to generally describe the execution of instructions/operations at a specific moment in time, based on a certain requirement. However, the concept of real-time must be described in depth especially in the context of communication protocols and synchronization of communication exchanges. The synchronization of operations and task recurrence are part of a real-time system, and controller applications are implementing these concepts, but in the context of communication protocols, the term should imply also specific capabilities. One of these capabilities is targeting the Quality of Service and the ability to assure the user that time intervals are managed efficiently, transmission and receiving procedures are guaranteed to be executed in the desired time interval, and not just from the timing point of view but also regarding the protocol capabilities. So, specifications regarding how procedures must be implemented and regarding the minimal necessary time of execution, must be placed in the same context. Based on this wide, context-targeted specifications, the user must be assured that mechanisms that are monitoring described aspects in the specification are in place and are also capable to react and survey the described operations at all time.

A second capability regarding the real-time context is the predictability of the system and fail case identification. Based on specifications, for a communication protocol to make the statement that is real-time oriented, it must provide identification of fail cases for both time and information volume, also taking in consideration interaction with different layers and interaction with required/demanded technologies.

The third capability of a communication protocol with real-time potential should imply safety mechanism for different scenarios and fail cases. Any system can fail eventually, even if the failure expectation is minimal, but when the failure appears, in the context of predictable behavior of the process, the user must have access to safety mechanism that reduces the damages, saves the status of the system at the fail moment and identifies and reports to the user the possible causes of failure, passed or not passed required time limits for different processes, steps to make to ameliorate fail situations and prevention guidelines.

In industry and production, having these capabilities translates to having the cost of maintenance and improvement kept at a low level, the down-time will decrease significantly, evolution of existing architectures will be done with higher awareness and prediction of risks and costs will be calculated in a more accurate manner. Having real-time capabilities should provide these types of advantages so the technology involved (communication protocols and network capabilities, for current scenario) should provide all necessary features to develop and maintain systems in an as described real-time context.

In the automotive domain, the real-time requirements are deterministic to most of the operations and are highly significant on different layers of the AUTOSAR Standard. Based on such requirements, from safety modules to powertrain and adaptive application, the software that runs on vehicles ECU’s is assuring high precision synchronization between processes and devices. Dealing with controller to controller transmissions over numerous busses inside the vehicle, interactions between software components, processing of data provided from a variety of sensors and multiple hardware with different capabilities involved in the infrastructure, the real-time requirements and implementations of solutions and strategies, as well as good practices based on specific use cases, have reached a very detailed level with years of technology and hardware evolution. With the adaptive platform being responsible to provide solutions for autonomous driving, use cases related to safety standards, over the air communication and high efficiency transmissions between different protocols, have achieved new standards regarding predictable behavior, time manipulation and synchronization, fault detection and safety measures. Based on the [26] requirements regarding fault detection are described with possibilities of error detection and resynchronization of time bases, certifying the need of safety mechanisms for different scenarios and fail cases in the context of real-time adaptive applications. Besides the detection of anomalies in specific use cases, dynamic subsystems responsible for countermeasures are also described and required for cases of desynchronization. Based on the same document, the importance of the definition of time is explained in the context of tasks executions and synchronization on Software Component level and also on ECU to ECU level. In the current implementation, the synchronization of receive and transmit operations on the Gateway Application have been made taking in consideration the details mentioned above regarding the execution of a task based on the timing of another task, and also the sync of tasks between different entities have been made, but not based on a common time base, each entity having a specific time base, the aim being to fulfill real-time requirements as close as possible to industry principles.

OPC UA Publish Subscribe mechanism was designed in the context of real-time requirements with focus on controller to controller communication, and easy integration with cloud architectures. Real-time publishing capacities have been achieved at high rates as stated in [27], the real-time synchronization of the process being managed at interrupt level. In the current implementation, the Subscribe process of the Gateway application aimed to comply with real-time behavior on application level and it have been used as part of a periodic exchange between an OPC UA Publisher responsible of distributing network messages regularly, with the final objective being for the VSOME/IP Client to receive information without missing any transmission cycle from the OPC UA Publisher.

## 3. Case Study and Results

**Figure 2:**
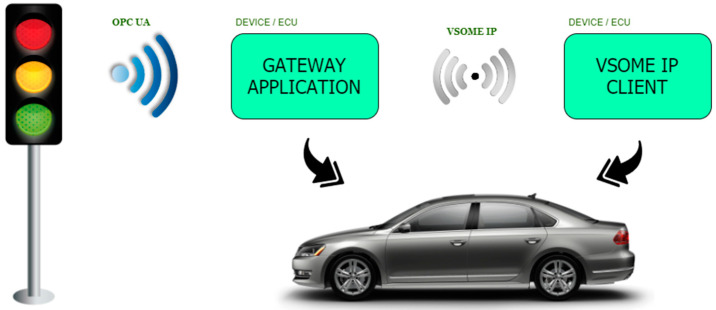
Case study general architecture.

Although the current work highly extends the gateway idea with several concepts and developments especially in OPC UA, the main scenario that was targeted with the current implementation considers that VSOME/IP is used in automotive, being used for intra-car communication, and OPC UA is usually used in infrastructure. The scenario regarding the interfacing of the vehicle with the infrastructure (in the current case a semaphore) can be observed in Figure 2, the objective being the development of a fast system that could make the information exchange possible and accurate at different time intervals.

### 3.1. Case Study 1

The semaphore would be represented trough an OPC UA server that could hold information needed by the vehicle. For the current study, the main information stored in the server were the colors of the light and the coordinates of the semaphore as a way of distinguishing a certain semaphore if more were to be involved in the traffic.

The OPC UA server has three nodes, one stores the current color of the semaphore, one stores the longitude, and another stores latitude. The color of the light is changed by an OPC UA client that runs in this case on the same machine as the server and uses Linux timers to identify the moment when the color should change. A way to test the concept is to have a third client that reads the data from the server, displays it and depending on the timestamp used for changing the values in the node destined to store the color, a user can regularly check the functionality of the concept. The bidirectional communication is achieved in a different manner than in [23], not by sending acknowledge messages (it is not desired in the current scenario to write a different value in the node by the gateway application), but by changing the value associated to the light of the semaphore at a certain period of time and only reading the values from the server with the gateway application. The OPC UA server will have the IP of the device assigned trough a hostname and the connection to the server can be done either with that IP for any device inside the same network, or with the IP of the router that manages the network for devices connected to the same network or connected to different networks.

Regarding the implementation of the OPC UA Server, the first step is to define the attributes needed for storing data using the UA_VariableAttributes Data Type. After a set of parameters are configured (description, datatype, access level), for each attribute, a hex value is assigned correspondent to the meaning of the information (color, longitude, latitude) using the UA_Variant_setScalar method. After all three attributes are configured and all values are assigned, the next step is to declare and configure the nodes, and introduce them in the information model using UA_Server_addVariableNode method. Afterward the server can run and the information will be available for the desired clients.

The next link in the information exchange is the gateway application that will read the information from the OPC UA server and send it to the VSOME/IP client. As in [23], there are two modules that are part of the gateway application. The first module is the OPCUA client, which reads the information from the OPC UA server following the Publish-Subscribe mechanism with UDP protocol on the transport layer. The OPC UA server can be accessed in the case study based on IP of the server, and the information is extracted from the nodes through browse procedure. The second module is the VSOME/IP service that will take the values received by the OPCUA client and prepare them to be sent to the VSOME/IP client by creating a specific payload. A different pattern that the one used by the authors in [23] was used for VSOME/IP communication. For the current study, the Notify-Subscribe mechanism was chosen, providing a faster and more efficient exchange of data between the VSOME/IP service and client. In [23], the bidirectional communication was achieved by exchanging acknowledge messages between the VSOME/IP client and service, process that is not needed when using the Notify-Subscribe pattern. For the communication to be made over the air, some network inputs are necessary for the gateway application, such as IP of the device, netmask, port, and others. Those inputs are stored in a .json file that is written in a specific way for the application. The .json file is assigned to the gateway application using VSOME/IP specific environmental variables.

The Implementation of the OPC UA Client side of the Gateway Application is done by initiating a connection with the server. After the connection is realized, a browse request is created and configured to return desired content from the Server using UA_Client_Service_browse. Another filtering based on the type of the nodes can be performed if not all received data is needed. The extracted payload is stored locally and gets prepared for transmission to the VSOME/IP Client. The Reading from the OPC UA Server and the transmission to the VSOME/IP Client are synchronized with a common timer, so predictable behavior is obtained and cyclic transmissions for the SOME IP Notify-Subscribe mechanism are achieved. However, in different scenarios, the reading from the Server can be desynchronized from the Transmission if multithreading or 2 step transmission is needed based on different data. The mechanism of browse request and browse response (UA_BrowseRequest and UA_BrowseResponse), facilitates the filtering of information from different nodes based on the node type and based on the information structuring. This ability allows future development of complex architectures for OPC UA Servers, architecture that can store huge amount of information without the risk of mixing it, or the need to transmit more data that is necessary to the clients involved in the transmission. This applies also when the client reads data from multiple servers. Having the browse service for traversing the address space and apply different filters, makes the client to build a hierarchy of nodes and it is usually used in the industry to extract metadata like properties of different variables and it is similarly used for the current concept. However, The Publish-Subscribe mechanism facilitates a more robust solution for filtering the information in a more abstract way and also makes the integration in an application with real-time requirements easier by providing information at a known moment in time. For initializing a valid browse request, the ua_browserequest_init function is used and sets the fields of a ua_browserequest structure with valid values. After the browse operation is finished, the allocated resources for the ua_browserequest structure must be cleared. The ua_browserequest_clear function frees allocated memory for the fields of a ua_browserequest structure and resets certain values for a future use, but the structure itself is not freed after the function is called, for that should be done separately based on specific requirements of the application.

In the context of the OPC Server-Client communication, the information is accessible for both the Client side of the Gateway application and also for the real-time Client that modifies the values from the Server by individual readings at different moments in time. The Client browses through the nodes and extract the information from the server different from how the Subscriber would receive information if the information would be published at different preconfigured recurrences. In the case of the Publish-Subscribe, the Subscriber gets information periodically from the network, and the browse is done internally for filtering useful and not useful data for that particular subscriber. Besides the pooling that the Client needs to do, another advantage for a Publish-Subscribe pattern would be that a Subscriber could receive information from different topics at different moments in time by subscribing to multiple DataSets, making the process easier than browsing constantly through the nodes of a Server. The VSOME/IP Service part of the Gateway Application set up the event group and starts sending the messages on the desired transport protocol, based on the configuration file. The payload of the transmission is based on the received values from the OPC UA Server. The cyclic time of the VSOME/IP Publisher is set at the same recurrences as the cyclic time of the OPC UA Publisher, so that for every receiving step in the Gateway application, a transmission is set to be initialized.

The Notify -Subscribe pattern of the VSOME/IP is an event-based mechanism that allows information exchange between A publisher and a subscriber via the VSOME/IP service discovery. The communication is done by so-called communication endpoints. The communication endpoints establish the transport protocol used, and other configuration parameters as port number, multicast address, and protocol specific details. All needed parameters are stored in a .json format in a VSOME/IP configuration file.

A subscriber entity can only subscribe to event groups, not to single events, with the help of the service discovery. The Server (Publisher) entity involved in the exchange, broadcasts messages containing all provided services, the Client application can transmit specific messages for subscribing to certain event groups. Service Discovery is used for detecting service instances, and to check if the handling of Publish/Subscribe mechanism is implemented. The Service Discovery messages are transmitted over UDP for the client entities to identify what services are offered.

The last link in the information exchange is the VSOME/IP client. By using the Notify-Subscribe pattern the client subscribes to the gateway application service that provides the values from the OPC UA server and it gets notified after every value received from the semaphore. The VSOME/IP client represents the destination of the whole process so the data that is received should be used for local process and control of different modules inside the vehicle. In a real case scenario, the latitude and longitude values would represent a mechanism to check if the information from the correct semaphore was read and the value that represents the color of the light should be an input for the ECU’s involved in the autonomous driving process. The VSOME/IP Client Subscriber is subscribing to the Published event group from the Gateway application and after the connection is initialized, information will be received periodically as soon as the information is published. Same as in the case of the VSOME/IP service from the gateway application, the configuration of the VSOME/IP client is done using a .json file in which the IP of the device is assign to the application, alongside with other network inputs. Also, the .json file is assigned to the application using environmental variables specific to VSOME/IP in the same manner as for the gateway application.

The data exchange was tested at different recurrences using a cyclic timer from the gateway application, timer that controls the process of reading the values from the OPC UA server and the process of transmitting the values to the VSOME/IP client right after the values were received. The recurrences used for the data exchange are from 1 ms to 10 s. The data received by the VSOME/IP client was 100% correct at any of the mentioned recurrences for this use-case. However, the latency of the transmission could grow depending on the amount of data desired to be transferred from OPC UA server to the VSOME/IP client in “one shot”, maintaining the accuracy of the received data.

The use-case was tested with all three links connected to the same network, and with OPC UA server connected to a different network than the Gateway application and VSOME/IP client. The behavior, speed of transfer, and accuracy of received data were identical.

### 3.2. Case Study 2

**Figure 3:**
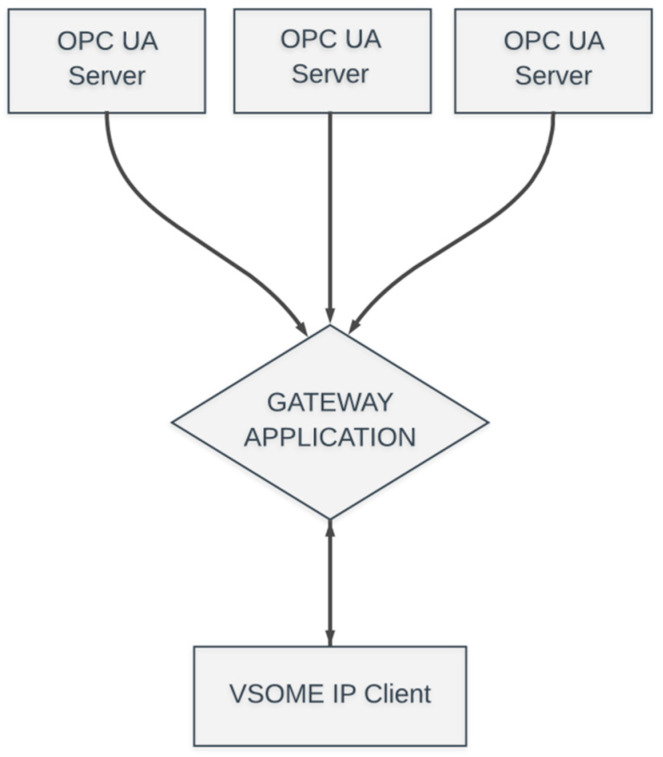
Second case study with the gateway accessing three different OPC UA Servers.

A second case study was implemented for the concept in a scenario where the gateway application is reading data from three different OPC UA Servers at the same time (see Figure 3). The scenario was meant to display the potential of interacting with multiple infrastructure entities represented by OPC UA Server. The objective was to achieve a speed of the transmission that could match the same recurrences as mentioned before and the received information to remain 100% accurate. The use case was tested as before, with all entities in the same network and also with each one in different networks. The data received by the VSOME/IP client were 100% correct at any of the recurrences used for this use-case (from 10-s transmissions to 1-ms transmission). A disadvantage represented by the interaction with multiple servers is the connection to all of them. Establishing the connection to more servers will increase the latency.

The connection to each server for any transmission cycle to the VSOMEIP final client, needs a variable period of time. Besides the connection itself, the browse operation through the address space of the server and identifying the desired information can produce variable latency based on the complexity and on the way that the information is structured in the server. In the case that there are problems with the connection, either network problems or security checks there is a timeout established before the connection is declared failed. In special cases where some servers may be in different networks or they may have additional security layers, the time before the connection is made can vary, so a latency may occur in the current study case. If the server also has a high volume of complex information and that information needs to be extracted from different places in the address space by the browsing operation, the latency mentioned early may increase in a variable way.

In the current scenario where the Gateway Application has to connect to multiple servers and extract and structure the information for each transmission to the VSOMEIP Client, in the case that additional connection steps are added (connection to additional servers), latencies may occur on both the connection and authentication part between the Gateway Application and servers and also on the browsing part necessary for extracting the desired information, latencies that may prevent the achievement of the general time criteria adopted for the current case study.

### 3.3. Case Study 3

The implementation for the third case study is based on the Publish-Subscribe paradigm of OPC UA, meaning that the information from the infrastructure (semaphore) is represented by an OPC UA Publisher, the Gateway receiving component is represented by an OPC UA Subscriber and that real-time requirements can be taken into consideration (also in the context of TSN technology) obtaining high speed transmissions and increasing feasibility of the application. The targeted requirements for this study case remained similar to the other study cases, the focus being the implementation of the most efficient paradigms for both OPC UA and VSOME/IP and the integration of those mechanism in a robust solution closer to a real use case.

For the current study, the first major challenge was to implement an OPC UA Publisher entity and an OPC UA Subscriber entity, that communicate on a multicast address and exchange a DataSet based on variables values. In the current context, the Publisher runs on a Linux-based system and publishes messages at a predetermined recurrence.

Once all the necessary components of the Publisher are configured, the information becomes available for the Gateway application that receives the data through the OPC UA Subscriber component and prepares it for being sent over VSOME/IP protocol to the target receiver. The VSOME/IP target receiver is constantly waiting to be notified by the Gateway application. The high rate and accuracy of transmission, and multiple data filtering and comparison options at different stages of the transmission, are powerful tools for a real case scenario involving the automotive field and IIoT. As an example, in a case of image processing, using a solution that allows the processing and transmission of information based on a traffic camera from the infrastructure to a vehicle, the usage of multiple protocols with Publish-Subscribe patterns and synchronization capabilities would represent major challenges of the process. The Gateway application represents a solution that embodies efficient mechanisms for transmitting and receiving information, and also complies with real-time requirements at application level on all devices involved in the communication flow.

The current solution runs on (classic) Raw Ethernet but should be compatible with TSN technology, being able to guarantee more accurate and more fast cycles of transmissions with real-time behavior on application level and also on network level.

The first step in the implementation was to create and configure all entities described above. The Publish-Subscribe connection was done for both the Publisher and also for the Subscriber (part of the gateway application) based on the OPC Unified Architecture Part 14: PubSub specifications. After the parameters are configured, the Publish-Subscribe connection is added to the server using the UA_Server_addPubSubConnection method. The Publish-Subscribe Connection is configured for both Publisher and Subscriber only at the start of the application. With the connection being a deterministic component of the applications, once the parameters are set, only in cases of reconfiguration, the connection should be modified.

The WritterGroup on the Publisher side was created and added to the connection using the UA_PubSubConnection_addWriterGroup method. As explained in the OPC Unified Architecture Part 14: PubSub specs (chapter 5.2 DataSet), the DataSet can be formed from Variables, Events, Timestamps, and more, and can be configured to have more fields of information. In the current context the payload of data will be represented As Variable Values in the published DataSet.

As a transport protocol, UDP is the chosen option for this case. The Subscriber will receive network messages, and it will apply filters for extracting the payload. Once the payload is Extracted, The VSOME/IP service functionality of the Gateway application takes over, preparing the information to be transmitted to the target VSOME/IP Client for a second filter of processing.

**Figure 4:**
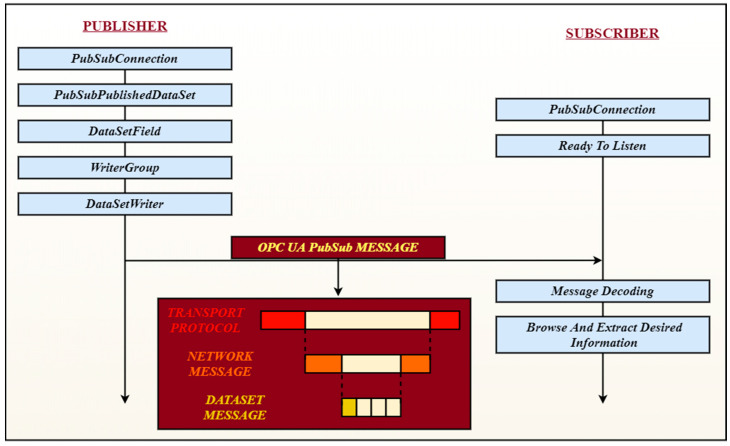
OPC UA Publish-Subscribe configuration components.

On the Subscriber side, after the Publish-Subscribe connection is done, The Gateway application will synchronize the receiving of the OPC UA message with the transmission of the information to the VSOME/IP Client. In Figure 4, the sequence of the necessary configuration for all components involved in the OPC UA Publish-Subscribe pattern can be observed, alongside different steps before and after the information is transmitted or received.

**Figure 5:**
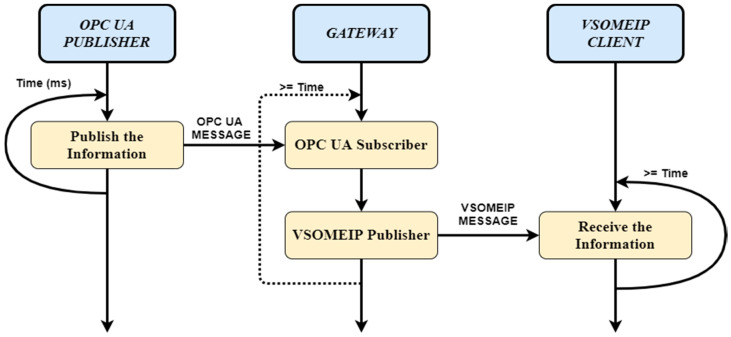
Dependency between Gateway application components, OPC UA and VSOME/IP in the Real-Time Publish-Subscribe context.

Using communication protocols and implementing the Publish-Subscribe mechanism, a correlation between publishing and receiving times for both protocols was necessary, alongside a dependency procedure between the Gateway application components (see Figure 5). The VSOME/IP Notifying (Publishing) process is dependent of the OPC UA Subscribing process so before the information arrives from the OPC UA Publisher, no Transmission is initiated to the Target Client, and after the information arrives from the OPC UA Subscriber and after the VSOME/IP message is transmitted, the execution sequence returns to OPC UA Subscribe process in a time, less or equal with the OPC UA Publishing time interval.

The OPC UA Publish-Subscribe mechanism was used successfully, and it accomplished the requirements of the use case.

### 3.4. Architecture

The general architecture of the concept is represented by 5 different entities with different goals:

1. OPC UA Server which stores data needed to be sent and represents the start point of the data exchange.
2. OPC UA Client which renews data in the OPC UA server at certain periods of time.
3. Gateway Application—OPC UA Client side responsible of reading data from the OPC UA Server.
4. Gateway Application—VSOME/IP service side responsible of sending data to a VSOME/IP Client.
5. VSOME/IP Client which represents the endpoint of the data exchange.

The main objective of the prototype is to make possible the communication over the air from the OPC UA Server, through the Gateway application, to the VSOME/IP Client, in the context of OPC UA Publish-Subscribe mechanism and VSOME/IP Notify-Subscribe mechanism.

**Figure 6:**
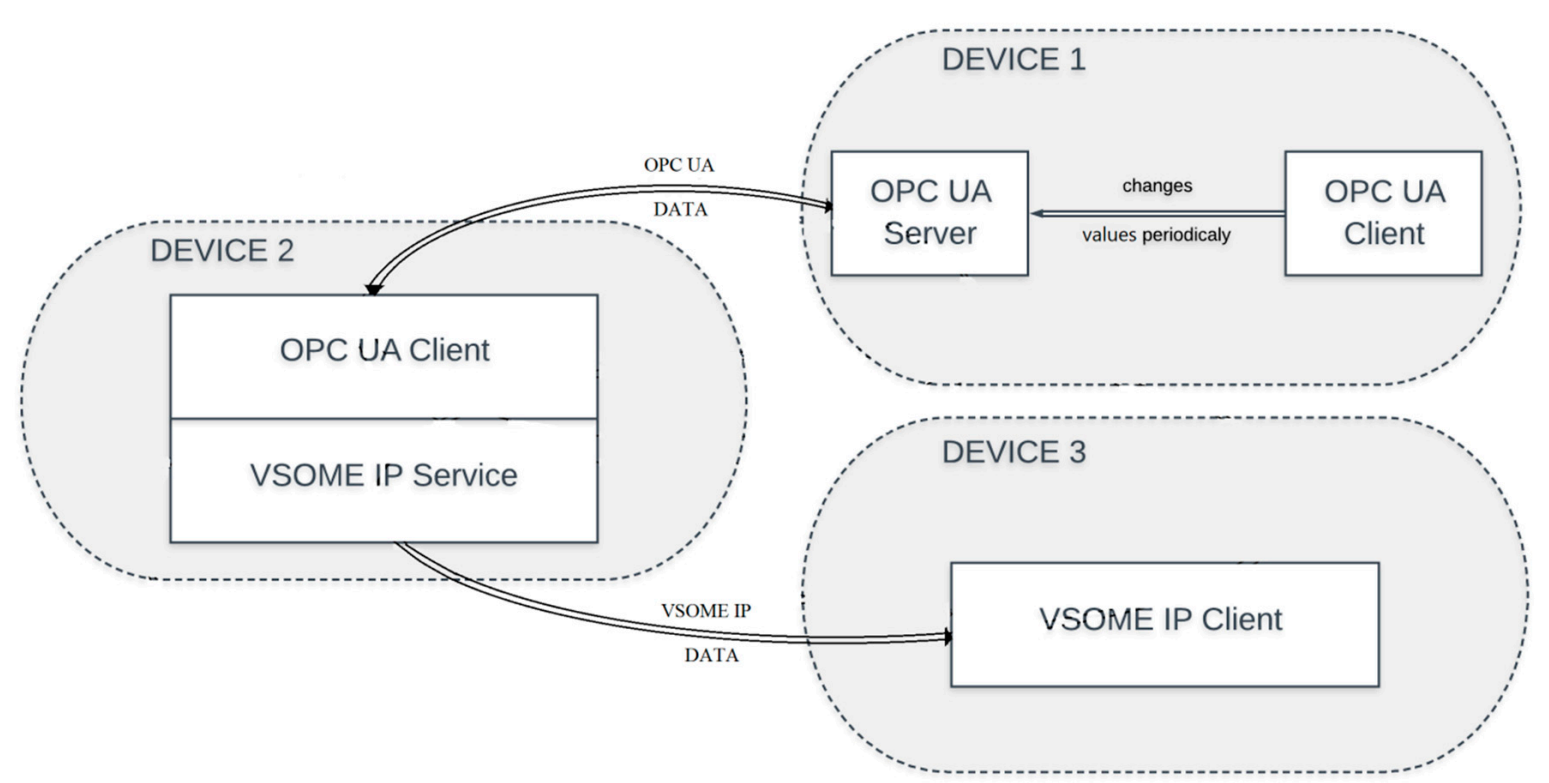
The three devices used in the case study architecture.

Each of the entities can run on any Linux-based device connected to a network. For a better view of the process three machines were used (see Figure 6):

- One where entities (1,2) are running;
- One where the OPC UA—VSOME/IP gateway is running, entities (3,4) components of the Gateway application;
- One where the VSOME/IP client runs, entity (5).

In the case of using the OPC UA Publish-Subscribe paradigm, the entities present on the first device are replaced only by the OPC UA Publisher and the OPC UA Client component of the Gateway application is represented by the OPC UA Subscriber.

### 3.5. Results

For each study case, beside the focus on the implementation of every involved entity, some general requirements were taken in consideration in terms of the time of the end-to-end transmission and efficiency. The fastest time interval targeted for all the study cases was 1 ms. Expected efficiency for each study case was 100%. In the case of a lower efficiency, the time interval should have been increased until 100% efficiency would be achieved. All study cases have proven successful in complying with the established criteria.

The prototype is designed based on a use-case where the data exchange is possible not only with the devices connected to the same network, but also in a manner where the Gateway application devices and VSOME/IP Client running network is different than the one where OPC UA server device is connected.

In Table 1, result of the case studies can be observed and compared based on the parameters that were taken into consideration by the authors in the development process of each version of the gateway application. The parameters regarding the time recurrence and efficiency have been applied for all study cases, even if only for study case 3 the used mechanism was developed to target real-time behavior. This approach was done for obtaining a solution closer to the intuitive real time requirements that may be present in a real context of V2I interfacing. For case study 3 the implementation complies with the OPC UA specification for the real-time oriented Publish-Subscribe mechanism and by also synchronizing the transmit and receiving the processes for all components and devices involved, the claim of obtaining real-time behavior at application level is achieved.

The implementation of the OPC UA Publish-Subscribe mechanism in the case study 3 has represented a more complex task in comparison with the other case studies, alongside the integration in the same context with the VSOMEIP Notify-Subscribe mechanism. Having to take into consideration the achievement of real-time behavior, the necessity to implement and configure all components necessary for the Publish-Subscribe pattern and the synchronizing of the receive and transmit operations between the protocols, are arguments for an increased complexity in implementation and testing. The implementation of case studies 1 and 2 is less complex in terms of architecture and configuration of the entities involved, compared to case study 3. The targeted use cases where communication have been tested from different networks represent an argument for an increased difficulty in implementation and configuration of the devices involved in the study. For the testing of all three case studies multiple servers and clients have been created and configured, task that increased the difficulty of testing the concepts.

With the real-time requirements at application level fulfilled, and using Ethernet at the data link layer, the solution proved efficient, guarantying the delivery of the information at different time intervals with minimal full transmission time of 1 ms and with 100% success rate regarding the payload integrity. It is expected for the capacity of the transmission speed to increase in the context of TSN, with Publish-Subscribe pattern being a key tool in the selection of high capability Communication Protocols and in development of Applications with real-time constraints, hardware flexibility and scalability demands.

## 4. Discussion and Conclusions

With the interaction of OPC UA’s Publish-Subscribe mechanism and the TSN technology in the context of IIoT, new capabilities are becoming available for various use-cases in the automation field. It is expected for future implementations and solutions based on the latest OPC UA’s specification to extend further the possibilities of real-time behavior of applications and to facilitate the interaction between different existing architectures. With the current solution, steps are made toward achieving a better understanding of the current relation between the needs of the industry and the suitable technologies, expanding future approaches to similar scenarios. In terms of time synchronization in messages exchanges over Ethernet topical mechanisms are used in similar ways to the industrial approach on the same matters, the development of the solution being based on concepts that represent a high interest in both automotive and automation fields.

The current study is expanding the knowledge in the area of interfacing OPC UA and VSOMEIP protocols in the context of Car to infrastructure communication by applying recent mechanism from both protocols and by integrating the implemented solutions in use cases of high interest. From the previous work in this area [23], the current work provides significant differences that improve the view on the capabilities of the protocols and the needs of the industry. The main differences are:

1. In [23] was a proof of concept for this type of gateway, the current work is a solution much closer to a real case scenario taking in consideration requirements present in the Automation Industry and Automotive.
2. In [23] other paradigms were used (for the OPC UA communication, the classic Server Client implementation was developed and for VSOME/IP the Request-Response mechanism was used). At the time of the development of [23], the latest OPC UA specifications were very new and for a proof of concept the usage of a more known mechanism in the case of OPC UA seemed a better approach.
3. In [23] the transmissions from different devices were not taken in consideration, the focus being only the proof of concept. In the current implementation the transmissions are made with each entity on a different device and also in some cases from a different network. Interaction with multiple OPC UA servers and clients was developed in the current work for proving the feasibility of such interfacing. All these details made the current implementation more complex than [23] in terms of architecture, development of each entity, and testing.
4. In [23] the real-time requirement was not even taken in consideration (it is specific to the Publish-Subscribe mechanism of OPC UA), in the current work, beside the analysis of what real-time behavior should mean for this type of applications, the implementation achieves a real-time behavior at application level by guaranteeing transmissions at different time intervals with the potential to achieve faster transmissions in the context of TSN technology.

In the context of the current work, the authors are concluding a brief comparison between OPC UA Publish-Subscribe and VSOME/IP Notify-Subscribe. VSOMEIP and OPC UA represent valuable solutions regarding fast and precise communication over a network and are highly used for multiple applications in Automotive and Industry. In the context of Internet of Things, where common goals must be achieved by both fields in regards of transmission capacity, synchronization and real-time behavior, scalability and easy integration with existing domain specific infrastructures, similarities and differences can be identified for the benefit of future industrial solutions responsible of shaping the interaction between vehicles and infrastructure, solutions that might consider to adopt the usage of both protocols in various and advantageous ways.

A primary advantage can be represented by the existing communication patterns for the both protocols. The request/response pattern specific to VSOMEIP is highly present in many applications and it can be portrayed as the classic exchange of messages between a Client and a Server, similar with OPC UA’s commonly used exchanges between Servers and Clients. The current architectures based on OPC UA are mainly using classic Client-Server transmissions, the latest Specifications regarding Publish-Subscribe concept being still recent, the industry is still relying on already implemented solutions with well-known results. However, the Publish-Subscribe pattern from part 14 of the OPC UA Specifications, is gaining popularity especially in the context of the time sensitive networks where new use cases are defined adding real-time behavior and easy integration possibilities in the current architectures. The Notify-Response pattern specific to VSOMEIP is describing a similar Publish-Subscribe concept used in similar ways for updating values or sending event notifications in applications with real-time requirements.

The integration and compliance of VSOMEIP with existing AUTOSAR architectures represent important achievements for real-time behavior, task synchronization, safety standards, and high usability. The industrial applications using VSOMEIP are developing high real-time performances such as message exchanges at nanoseconds level, being used for in-car communication in networks with high capacity and using a variety of hardware designed to operate in concordance with strict real-time requirements. OPC UA’s Publish-Subscribe mechanism was designed for controller to controller communication and the use of the concept in time sensitive networks can satisfy real-time requirements at some degree, however, with the evolution of the TSN technology, and with new, high scale applications being developed, the requirements will eventually extend, so for assuring full real-time behavior, future researches will be needed to identify new ways of development and improvement for OPC UA and other communication protocols that claim important roles in the Industrial Internet of Things.

## Figures and Tables

**Table 1:** Case study results analysis.

Case Study	Mechanism	Advantages	Disadvantages	Efficiency	Time Recurrence
**1**	OPC UA Server-Client	Moderate difficulty in implementation	Not real-time oriented	100%	≥ 1 ms
**2**	OPC UA Server-Client	Moderate difficulty in implementation	Not real-time oriented, could produce latency	100%	≥ 1 ms
**3**	OPC UA Publish-Subscribe	• Real-time oriented	• High complexity in implementation	100%	≥ 1 ms
		• Fast, reliable and close to technologies used in Automotive	• High difficulty in synchronizing processes on different devices without TSN Technology		
		• Potential to achieve faster transmissions with TSN Technology			
